# Nontraditional translation is the key to UFMylation and beyond

**DOI:** 10.1016/j.jbc.2022.102431

**Published:** 2022-08-28

**Authors:** Mengjia Lin, Xiaoyun Zheng, Jianping Jin

**Affiliations:** 1 Zhejiang Provincial Key Laboratory for Drug Evaluation and Clinical Research, First Affiliated Hospital, Zhejiang University School of Medicine, Hangzhou, Zhejiang, China; 2 Life Sciences Institute, Zhejiang University, Hangzhou, Zhejiang, China; 3 Center for Life Sciences, Shaoxing Institute, Zhejiang University, Shaoxing, Zhejiang, China; 4 Cancer Center, Zhejiang University, Hangzhou, Zhejiang, China

**Keywords:** Ufm1, UFMylation, de-UFMylation, UFSP, near-cognate start codon, cDNA, complementary DNA, Ufm1, ubiquitin-fold modifier 1, UFSP, Ufm1-specific protease

## Abstract

The Ubiquitin-fold modifier 1 (Ufm1) is a ubiquitin-like protein that can also be conjugated to protein substrates and subsequently alter their fates. Both UFMylation and de-UFMylation are mediated by Ufm1-specific proteases (UFSPs). In humans, it is widely believed that UFSP2 is the only active Ufm1 protease involved in Ufm1 maturation and de-UFMylation, whereas UFSP1 is thought to be inactive. Here, Liang *et al.* provide strong evidence showing that human UFSP1 is also an active Ufm1 protease. These results solve an age-old mystery in the human Ufm1 conjugation system and could have a greater impact not only on Ufm1 biology but also on the translation of genes employing nontraditional start codons.

Ubiquitin-fold modifier 1 (Ufm1) is a ubiquitin family protein that can be conjugated to its protein substrates in a process termed ‘UFMylation’ *via* a classic E1-E2-E3 enzymatic cascade (1, 2, 3, 4). Like many other ubiquitin family proteins, Ufm1 is translated as a precursor, the C-terminal dipeptide of which must be cleaved to yield mature Ufm1, which is subsequently activated by the Ufm1-activating enzyme Uba5 to initiate UFMylation (1, 2). Like protein ubiquitination, neddylation, or SUMOylation, protein UFMylation is also a reversible process (2, 5).

Both Ufm1 maturation and de-UFMylation are mediated by Ufm1-specific proteases or UFSPs (5). UFSPs are encoded by two genes in humans and other species (5); however, it is widely accepted that UFSP2 is the only active Ufm1 protease that performs these processes in human cells; in contrast, human UFSP1 is considered as an inactive protease, since it only has partial sequences of the protease domain, whereas in other species UFSP1 does contain the whole protease sequence. Yet, the unique evolution of human UFSP1 has long remained a mystery.

A recent discovery by Liang *et al.* (6) showed some rather surprising results. Initially, they noticed that UFMylation was enhanced when UFSP2 was knocked out in human cells, suggesting that there is an additional protease involved in the maturation of the Ufm1 precursor. Then, they immediately turned to UFSP1. They first expressed the full-length complementary DNA (cDNA) of UFSP1, including its 5′-UTR, and found that it could de-UFMylate Ufm1 conjugates in 293T and HeLa cells. This cDNA produced a ∼23 kDa protein, instead of the ∼17 kDa protein, predicted based on the canonical open reading frame (ORF) that showed no de-UFMylation activity when transfected in cells. Using a reverse genetic approach, the authors also noticed that UFMylation was slightly enhanced in UFSP1 KO cells but strongly increased in UFSP2 KO cells, suggesting that UFSP2 could be a major protease involved in de-UFMylation of substrates. When both proteases were knocked out, no UFMylation was detectable. These results strongly indicated that UFSP1 employs a noncanonical ORF to produce a Ufm1 protease mainly responsible for the maturation of the Ufm1 precursor. Evidently, the canonical ORF only contains part of the coding region of the full-length UFSP1. The authors concluded that human cells produce an active UFSP1 from a nontraditional start codon instead of at a classic AUG codon and that its main function is to make mature Ufm1.

Besides the cognate AUG start codon, eukaryotic cells also employ other near-cognate start codons to initiate translation (7, 8). For example, the oncogene c-*myc* uses both near-cognate CUG and cognate AUG as translational initiation codons to synthesize c-*myc*1 and c-*myc*2 oncoproteins, respectively (9). In fact, the most frequent near-cognate start codon is CUG. Because the full-length cDNA produces a ∼23 kDa protein, Liang *et al.* selected four candidate CUG codons based on predicted molecular weights of polypeptides and their abilities to synthesize active UFSP1 enzymes. Using serial mutagenesis-based approaches, the authors found that ^217^CUG is the only potential start codon. To provide even more evidence, they turned to mass spectrometry. The authors engineered a FLAG-tag at the carboxyl terminus of UFSP1 using a CRISPR/Cas9-based knock-in method and then purified the UFSP1-FLAG fusion proteins from engineered cells. Six peptides covering more than 90% of the predicted N-terminal peptide sequences from ^217^CUG to the canonical ^445^AUG codons were identified using this method. In addition, using *in vitro* assays with purified recombinant proteins, the authors further consolidated UFSP1 as a protease in the maturation of Ufm1 precursor and de-UFMylation. Even more surprisingly, they provided some evidence suggesting UFSP1 as an even stronger enzyme for both Ufm1 maturation and de-UFMylation of Ufm1-conjugated ASC1 compared to UFSP2.

CUG is the most common near-cognate start codon employed in eukaryotes, but the mechanism behind its use is still unclear. The authors of this study discovered that the 5′-UTR sequence is essential for UFSP1 expression in humans; in particular, they identified an E-box motif, CAGCTG, which plays an important role in human UFSP1 expression. Furthermore, they confirmed the critical role of the eukaryotic translation initiation factor eIF2A in translation of UFSP1 from the CUG codon using both chemical and RNAi approaches.

Ultimately, Liang *et al.* (6) provided strong evidence that UFSP1 is an active Ufm1 protease in human cells and that its translation is initiated at a near-cognate CUG start codon. Moreover, they identified an E-box sequence at the 5′-UTR and eIF2A as critical factors in human UFSP1 expression. However, there are still some unanswered questions that remain. For example, are there any RNA-binding proteins that can specifically recognize the E-box sequence to assist the CUG start codon usage? If so, how can these proteins coordinate with eIF2A to initiate UFSP1 translation at the near-cognate start codon? Also, the authors claim that the enzymatic activities of UFSP1 are higher than that of UFSP2 in *in vitro* assays, although it appears that UFSP2 is better at de-UFMylation in cells. The question is, does the recombinant UFSP2 protein lose some activity during the purification process? Would the HA-tag sequence, if fused at the carboxyl terminus of UFSP2, lead to stronger cellular activity when expressed in cells, since the localization of UFSP2 to the endoplasmic reticulum membrane is potentially critical for its maximal activity? Furthermore, the authors only used ASC1 as a substrate in their assay, but these two proteases might behave differently toward other Ufm1 substrates. It is worth mentioning that a similar study by Millrine *et al.* (10) answered part of this question. Finally, does the E-box sequence discovered here exist in the 5′-UTR of other genes which employ near-cognate start codons as translation start sites? Nevertheless, the discovery of an active UFSP1 opens a new avenue to study biological functions of UFMylation in humans. The identification of the E-box sequence could also provide clues to the mechanisms behind near-cognate translation initiation. If so, this discovery could have a much broader impact beyond studies of UFMylation.

## Conflict of interest

The authors declare that they have no conflicts of interest with the contents of this article.
